# Morbidity associated with "self-rated health" in epithelial ovarian cancer survivors

**DOI:** 10.1186/1471-2407-9-2

**Published:** 2009-01-02

**Authors:** Astrid Helene Liavaag, Anne Dørum, Sophie D Fosså, Claes Tropé, Alv A Dahl

**Affiliations:** 1 Department of Gynecology, Sorlandet Hospital, N-4806 Arendal, Norway; 2 Department of Gynecological Oncology, The Norwegian Radiumhospital, Rikshospitalet University Hospital, N-0310 Oslo, Norway; 3 Department of Clinical Cancer Research, The Norwegian Radium Hospital, Rikshospitalet University Hospital, N-0310 Oslo, Norway; 4 Faculty Division The Norwegian Hospital, University of Oslo, N-0316 Oslo, Norway

## Abstract

**Background:**

Epithelial ovarian cancer survivors (EOCSs) frequently report multiple complaints after their treatment. The objective was to study somatic and mental morbidity in EOCSs associated with their Self- Rated Health (SRH) assessed by a single item.

Findings were compared to age-matched controls from the general population.

**Methods:**

In a cross -sectional follow-up design 189/287 (66%) EOCSs treated at The Norwegian Radiumhospital 1979–2003 responded to a mailed questionnaire on demographic data, and somatic and mental morbidity. SRH last week was rated on item #29 of the European Organization and Treatment of Cancer Quality of Life Questionnaire in 84/189 (97%) of responding EOCSs. For comparisons "good" and "poor" SRH groups were defined by the median score on the SRH item.

**Results:**

EOCSs with "poor SRH" had higher level of somatic symptoms, anxiety, depression and fatigue than those with "good SRH" (p < .001). In multivariate analyses somatic symptoms, age and fatigue, were significantly associated with the SRH score in EOCSs, but not the cancer-related variables (FIGO stage, recurrence in < 6 months or chemotherapy ever). The model explained 70% of the variance in SRH in linear and 77% in logistic regression analyses. The distribution of the SRH scores in EOCSs did not differ significantly from that of normative controls; however a higher proportion of controls recorded a high SRH score.

**Conclusion:**

SRH is strongly related to common somatic complaints, impairment and fatigue but not to cancer-related variables. A single question concerning SRH last week might be a quick screening method for collecting important information on symptoms in EOCSs, in addition to cancer – related questions.

## Background

Due to successful multimodal treatment the number of women who survive epithelial ovarian cancer has increased over the last decades. The 5-year survival rate is 45% in Norway, ranging between > 80% in stage I of the International Federation of Gynecology and Obstetrics (FIGO) classification to < 20% in FIGO stage IV [1,2]. The age-adjusted incidence rate of ovarian cancer is 14 per 100,000 women yearly in the United States and 11.8 per 100,000 women in Norway. Bilateral- oophorectomy (BO) is the primary treatment of epithelial ovarian cancer combined with chemotherapy dependent on the FIGO stage. [1,2], however, the majority experience several relapses and are repeatedly treated with chemotherapy.

Several studies have shown that epithelial ovarian cancer survivors (EOCSs) have significantly higher levels of somatic and mental symptoms as well as fatigue compared to women of the general population [3-6]. Stewart et al. [3] reported that among 200 EOCSs without evidence of disease for two years, 98% regarded their health as good or excellent, though 54% had current pain or discomfort. We have previously shown that compared to controls, EOCSs > 18 months after primary treatment had significantly more chronic fatigue, somatic and mental morbidity, somatic complaints, use of medications and more frequently used health care services than population-based controls [6]. After treatment, EOCSs will repeatedly consult their gynaecologists and their regular general practitioners (GPs), who will assess the EOCSs' performance and health status by multiple questions in addition to physical examinations and laboratory tests. On the other hand, a single statement about self-rated health (SRH) allows the patients to express various aspects of their health in a global statement. If considered as "poor", more focused follow-up questions may be raised, identifying symptoms which may be accessible for therapeutic intervention. If SRH is stated as "good", no further investigation is needed at the moment [7,8]. There is a widespread consensus that a simple, global SRH questions may provide a useful summary of the patients' overall health status, and SRH has thus been considered as an important measure of treatment outcome [7-14]. A frequently used measure of SRH is a single question asking patients to rate their overall health on a scale from excellent to very poor, and there are many alternative phrasings of such a global question [7-14]. Two questions of the European Organization and Treatment of Cancer (EORTC) Quality of Life Questionnaire (QLQ C-30) [15,16] have been used in various SRH studies: item #29 "How would you evaluate your overall health during the past week?" and item #30:"How you rate your overall quality of life during the last week". Both items are scored on 7 point Liker scales, and the responses to these two items are highly correlated. The literature, however, has shown that overall health relates principally to somatic problems, while quality of life encompasses mental health to a greater extent [7,8].

Butt et al. [9] found single-item screening questions to be quite effective for fatigue, pain, distress and anorexia in ambulatory cancer practice with a mixed sample of cancer patients. Also Rohrer et al. [17] showed in their cross sectional study of 1,183 patients in five community clinics that the use of a single – item measure of SRH proved to have clinical value in primary care and helpful to identify a number of somatic, metal and quality of life symptoms. Shadbolt et al. [10] reported that SRH responses were valid, reliable and responsive to objective change of health in patients with advanced non-gynecological cancer. Al-Windy et al. [11] investigated SRH in a general population based sample of 470 adult persons of multi-ethnic origins who visited a Swedish Health Care Centre. On the basis of SRH the authors grouped the persons into a poor and a good health group, and found SRH helpful to identify the 46% of the patients who had a significant number of symptoms. Eriksson et al. [12] in a random sample of 8,200 Swedish persons compared three different SRH questions in order to find the best question measuring lifestyle, psycho-social, mental and physical health and found this a useful method. The results imply that the different measure represents parallel assessments of subjective health and that measures without specified response options are better in younger than older population groups.

To our knowledge there are no studies of SRH and its associations to somatic and mental morbidity in EOCSs. We therefore wanted to: 1) Explore the somatic, mental and lifestyle variables associated with a single question concerning SRH (EORTC) (QLQ C-30) item #29 "How would you evaluate your overall health during the past week?" for therapeutic use in a clinical setting. 2) Explore to what extent the SRH score of EOCS differ from those of age-matched women in the general population (NORM).

## Methods

### Patient selection

The EOCSs were 20–70 years at survey with > 18 months survival since diagnosis. They had been treated according to protocols for FIGO stage I-III epithelial ovarian cancer (EOC) at the The Norwegian Radium Hospital [18]. In order to get a sufficiently powered sample size, we had to include cases back to 1979. The inclusion criteria were fulfilled by 297 EOCSs who were alive by September 2004, and who received a mailed questionnaire. Ten cases were subsequently excluded after revision of histology, leaving an eligible sample of 287 EOCSs. A total of 184/189 (97%) EOCSs returned the questionnaire with valid rating concerning SRH, and had basic anthropometric measures and blood samples taken at their regular general practitioners (GPs). One reminder was sent to non-responders after four weeks.

### Treatment principles

All EOCSs had primary surgery with BO, and the majority also had hysterectomy, omentectomy and maximum debulking. Primary treatment was done according to established protocols, as either surgery only, or combined with subsequent chemotherapy depending on FIGO stage, histology and ploidy [19].

Platinum-based chemotherapy represented the most frequent systemic treatment. Paclitaxel was incorporated into the combination chemotherapy in the 1990-ies [18]. The majority of EOCSs received combined carboplatin and taxol as first line treatment, and nine patients got cisplatin monotherapy. Relapses were treated with various types of chemotherapy administered as combinations or mono-therapy. Among the drugs used were paclitaxel, carboplatin, gemcitabine and tamoxifen, and four EOCSs also had local radiotherapy.

### Measurements

#### EORTC QLQ-C30

The EORTC QLQ-C30 [15] consists of 30 items comprising five functional scales, an overall Global health scale/QOL scale, three symptom scales, and six single symptom items. The scores are transformed to 0 – 100 scales: on the functional scale higher scores represent better functioning, while on the symptom scale higher scores mean more severe symptoms. In this study the presence of somatic complaints was defined by the median scores (for nausea and pain a score ≥ 16.67, for dyspnoea, insomnia, constipation, lack of appetite and diarrhoea a score ≥ 33.33).

Item # 29 had seven score alternatives and was considered to reflect SRH. We transformed the 1 to 7 response score to 0 to 100 and used the median score (66.67) to separate the EOCSs into a "poor SRH" and a"good SRH" group. The correlation between item #29 and #30 was r = 0.83 with 69% explained variance in our EOCSs sample.

As EORTC QLQ-C30 has been psychometrically validated cross-culturally, all scales and single items have been tested according to standards for reliability [16]. The internal consistencies of the functioning scales were: physical α = .76, role α = .87, emotional α = .89, cognitive α = .72, social α = .87 and overall QoL/Global health α = .90.

#### The Hospital Anxiety and Depression Scale (HADS)

The HADS [19] consists of 14 items, 7 on the depression subscale (HADS-D) and 7 on the anxiety subscale (HADS-A). Each item is scored on a four-point scale from 0 (not present) to 3 (considerable), and the item scores are summarized for each scale, giving HADS-D and HADS-A scores. Based on the literature, cases of HADS-defined anxiety disorder or depression were defined by a score of ≥ 8 on HADS-A or HADS-D, respectively. Internal consistency of the anxiety subscale was α = .88, and of depression α = .84 in the EOCSs sample.

*The Fatigue Questionnaire (FQ) *consists of a total of 13 items, where 11 assess the presence and intensity of fatigue symptoms [20]. Seven items assess Physical Fatigue and four assess Mental Fatigue. Summarized they represent the Total Fatigue score. Based on Total Fatigue score and duration of fatigue ≥ 6 months, caseness of Chronic Fatigue (CF) are identified. Internal consistency was for physical fatigue α = .92, mental fatigue α = .68, and total fatigue α = .89 in the EOCSs sample.

*The Body Image Scale (BIS) *is a 10-items self-rating-scale developed to assess changes of the body image in cancer patients [21]. The BIS focuses on how EOCSs feel about their appearance during the past week and on changes in appearance due to her cancer and/or treatment. Each item is scored on a four point Likert scale: from 'not at all' (0) to 'very much very' (3). Increasing BIS score represents poorer body image. The internal consistency was α = .91 of the BIS in the EOCSs sample.

*The Intimate Bond Measure (IBM) *is a 24-item self-rating scale measuring experienced care and control from her partner in the patient's current relationship [22]. Both care and control are assessed with 12 items. Each item is scored on a four point Likert scale from 'not at all' (0) to 'very true' (3). Higher values mean more care or control. Among the EOCSs in paired relations the internal consistency for care was α = .96 and for control α = .92.

*The Menopause-Specific Quality of Life Questionnaire(M-QOL) *is a 29 items questionnaire covering the vasomotor, physical, psychosocial and sexual menopause-related domains [23]. The M-QOL was included since has been shown that menopausal symptoms can be present for a long time in EOCSs [24]. Each item is first scored as present or not, and if present a seven point scale of severity from 'not at all bothered' (0) to 'extremely bothered' (6) is filled in. In EOCSs the internal consistency for physical α = .74, psychosocial α = .61 vasomotor, α = .79 and sexual α = .46. Due to the low internal consistency the sexual domain was excluded from the analyses.

*The Sexual Activity Questionnaire (SAQ) *consists of three sections covering: 1) Relation status, 2) Reasons for sexual inactivity; and 3) Sexual functioning (SAQ-F) [25,26]. The SAQ-F has a time frame of last month, and consists of 10 items with three subscales: sexual pleasure (7 items), sexual discomfort (2 items) and change in sexual habit (1 item). Sexual pleasure and discomfort are rated on a four-point Likert scale from 'not at all' (0) to 'very much' (3). An increasing score means more pleasure and more discomfort, respectively. In the sexually active EOCSs the internal consistency for SAQ-pleasure was α = .88 and α = .78 for SAQ-discomfort.

*Education *was categorized into three levels based on the number of completed school years (≤ 10 years, 11–12 years, > 12 years). *Paired relation *described those married or cohabiting. Having *paid work *was defined as income from employment or independent business. *Level of physical activity *was dichotomized into "minimal", or "moderate or more" according to Thorsen et al. [27]. *Physical impairment and mental impairment *were defined as significant impairment in daily life for more than one year due to disease, injury or symptoms.

All *co-morbid conditions *were self-reported by responses to the general formulation: "Have your doctor ever said that you suffer from...?" The self-reported diagnoses were not validated by their regular GPs or hospital records. *Treated hypertension *was defined by current use of an antihypertensive drug, while current hypertension was defined by a blood pressure ≥ 140/90 mm Hg. *Hormone replacement therapy *(HRT) was reported as current use of sex hormones, while *analgesic *and *psychotropic *medication was reported as regular use last year. *Musculo-skeletal diseases *(osteoporosis, fibromyalgia, arthrosis, or other long-standing musculo-skeletal diseases) were defined as a diagnosis given by their GPs. *Daily smoking *concerned current consumption of any number of cigarettes.

A clinical Prognostic Index in EOCSs had been constructed by our group [6,28] based on scoring of established prognostic factors: age at diagnosis, FIGO stage, relapse within 6 months, modalities of primary treatment and duration of follow-up time. Presence of prognostic factors was scored as 1, and absence scored as 0, accordingly: age at diagnosis (≥ 50 years = 1, < 50 = 0); duration of follow-up (< 5 years = 1, ≥ 5 years = 0); FIGO stage (stage III = 1, stage I - II = 0); relapse within 6 months (Yes = 1, No = 0); and primary treatment modality (Surgery + post-operative chemotherapy = 1, surgery only = 0). The scores were added-up to an index sum score, and the EOCSs were allocated to a worst (score 4–5), medium (score 2–3), or best prognosis (score 0–1) group.

### The NORM sample

Normative data for the EORTC QLQ-C30 was obtained by the Cancer Clinic in 2004 [29]. Using public address lists, an anonymous age-representative sample of 3,500 Norwegian female population aged 20 to 79 years received a questionnaire with these instruments. Of the respondents, 1,267 (41%) were in the 30–71 years age range of the EOCSs, without cancer, and had completed the EORTC QLQ-C30. We randomly selected three controls for each EOCSs based on their distribution in 5-year age-groups from 30 to 75 years (NORM).

### Statistical analysis

Continuous data were analyzed by independent sample t-tests and categorical data with Pearson's χ^2 ^test. 95% confidence intervals (95%CI) were included as appropriate. Non-parametric tests were applied for skewed distributions. Internal consistency of a domain/subscale was examined with Cronbach's coefficient alpha. Statistically significant findings on continuous variables and on 2 × 2 contingency tables were also calculated as effect sizes (ESs) and values ≥ .40 were considered as clinically significant [30,31]. Relevant data were entered as independent variables in a stepwise hierarchical linear regression analysis with the SRH score as dependent variable. The strength of the associations was expressed as standardized final beta values with all steps in the model, and by explained variance (R^2^) and by change in explained variance (R^2^-change) for each step. The same procedure was done with the dichotomized SRH score using logistic regression analyses, however the strength of association were expressed as odds ratios (ORs) with 95% confidence intervals (95%CI).

The level of significance was set at p < .05 and all tests were two-tailed.

### Ethics

The study was approved by the Regional Ethics Review Board of South-Norway, and the Norwegian Data Inspectorate. All EOCSs provided written informed consent.

## Results

### Attrition analysis

An attrition analysis of the total number of EOCSs respondents (N = 189) and non-respondents (N = 98) showed that except for longer time since primary treatment among the respondent (mean 6.3 versus 4.4 years, p = .003, ES = .35), no significant differences in other clinical variables were observed. (Data not shown).

### Clinical characteristics of the EOCSs

SRH last week was rated by 184 of 189 responding EOCSs (97%). Among these 184 EOCSs the median age at diagnosis was 52 years (range 23–68 years), median age at survey 59 years (range 31–71 years) and median follow-up time 4 years (range 2–27 years). The FIGO stage distribution was: stage I 42%, stage II 18% and stage III 40%. Thirty-three percent of EOCSs had surgery only as primary treatment, while 67% also got chemotherapy. Using a five years limit since primary treatment, 44% were considered as long-term and 56% as short-term survivors. Relapse ever was observed in 31% of EOCSs. Sixty-five per cent had < 13 years of basic education, 57% had paid work, and 42% were pensioned, unemployed or on social support, and 75% were in a paired relation. (Table 1).

**Table 1:** Demographic and cancer-related characteristics of EOCSs in the "good" and the "poor" SRH groups.

Variables	Good SRH (N = 89)	Poor SRH (N = 95)	P	ES^a^
	*Mean (SD)*	*Mean (SD)*		

Age at diagnosis	52.7 (9.2)	50.2 (9.2)	.07	
Age at survey	59.3 (8.5)	56.2 (8.3)	.02	.37
Follow up time^b^	6.6 (5.9)	6.0 (6.1)	.54	

	*N (%)*	*N (%)*		

*Level of education*			.62	
< 10 years	23 (26)	25 (27)		
11–12 years	38 (43)	33 (36)		
> 12 years	28 (31)	34 (37)		

*Employment status*			.06	
Paid work	58 (65)	49 (52)		
Unemployed/pensioned	31 (35)	46 (48)		

*FIGO stage*			.60	
I	40 (45)	38 (40)		
II	13 (15)	19 (20)		
III	36 (40)	38 (40)		

Recurrence in < 6 month	25 (28)	33 (35)	.33	

Chemotherapy + surgery	62 65)	62 (70)	.35	
Surgery only	32 (36)	28 (30)		

*Prognostic index*			.35	
Good	26 (29)	26 (37)		
Medium	49 (55)	46 (48)		
Poor	14 (16)	23 (24)		

On-treatment last 6 month	25 (28)	33 (35)	.33	

^a ^Effect size. ^b ^Non-parametric test

### Comparison within the EOCSs group

#### "Good SRH" and "poor SRH" – demographic, cancer-related and somatic variables

According to the definition, 89 (48%) (95%CI 41–57%) EOCSs had"good SRH" and 95 (52%) (95% CI 44–59%) had"poor SRH". Mean age at survey was significantly lower in the patients with"poor SRH" (ES = .37), but no significant differences were observed for other demographic or for cancer-related variables such as FIGO stage, recurrence in < 6 months, chemotherapy ever, follow-up time or prognostic index. (Table 1).

Somatic complaints and physical and mental impairment were significantly more often reported in the "poor SRH" group compared to the "good SRH" group, and all differences except for diarrhoea had ES > .40, indicating clinical significance (Table 2). Both use of daily medication and use of analgesic and psychotropic medication last year were significantly more common in EOCSs with "poor SRH" compared to those with "good" SRH (ES = .46). The prevalence of somatic diseases did not differ significantly between the groups with "poor" and "good" SRH (Table 2).

**Table 2:** Somatic morbidity of EOCSs in the "good" and the "poor" SRH groups.

Variables	Good SRH (N = 89)	Poor SRH (N = 95)	P	ES^a^
	*N (%)*	*N (%)*		

Infarction, angina, stroke	16 (18)	23 (24)	.35	

Hypertension > 140/90				
Treated and untreated	31 (44)	31 (42)	.83	
On antihypertensive medication	13 (15)	19 (20)	.34	

Diabetes	1 (1)	4 (4)	.20	

BMI > 28	21 (25)	24 (27)	.74	

Hyperthyroidism/hypothyroidism	11 (12)	13 (14)	.79	

Musculo-skeletal diseases	33 (37)	44 (46)	.20	

Aches and pain > 3 months/last year	44 (49)	64 (67)	.01	.40

*EORTC somatic complaints*				
Nausea	1 (1)	17 (22)	< .001	.77
Pain	11 (19)	67 (81)	< .001	1.02
Dyspnoea	21 (23)	49 (52)	< .001	.93
Insomnia	37 (42)	75 (79)	< .001	.60
Lack of appetite	5 (6)	33 (35)	< .001	.77
Constipation	26 (29)	50 (53)	.002	.43
Diarrhoea	25 (28)	42 (45)	.022	.36

*Physical impairment*	7 (8)	46 (52)	< .001	1.10
*Mental impairment*	4 (5)	22 (25)	< .001	.70

*Physical activity*			.55	
Minimal exercise	23 (26)	20 (22)		
Moderate or more exercises	66 (74)	74 (78)		

*Medication last year*				
Daily use of medication	57 (64)	84 (91)	< .001	.46
Use of analgetics	6 (7)	35 (37)	< .001	.77
Use of psychotropics	13 (15)	47 (50)	< .001	.78

^a ^Effect size.

#### "Good SRH" and"poor SRH" – mental distress, sexual function, intimate relations, fatigue and quality of life

Compared to the "good SRH" group, the "poor SRH" group had significantly higher level of anxiety and depression, poorer body image, and higher levels of physical, mental and total fatigue, as well as lower M-QoL scores on the physical, psychosocial and sexual domains. All these differences were clinically significant (Table 3). A higher proportion of women with "good SRH" was sexually active (p < .001), and they scored higher on sexual pleasure (ES = .50) than those with "poor SRH". No significant differences were observed concerning sexual discomfort or current use of HRT (Table 3).

**Table 3:** Mental morbidity, intimate relations, sexuality, body image, fatigue and menopausal-related quality of life of EOCSs in the "good" and the "poor" SRH groups

	Good SRH (N = 89)	Poor SRH (N = 95)	P	ES^a^
*HADS*^b^				
Anxiety level, mean (SD)	3.8 (3.2)	7.1 (3.8)	< .001	.51
Depression level, mean (SD)	1.7 (1.8)	4.4 (3.7)	< .001	.92
Caseness of anxiety, N (%)	9 (10)	45 (47)	< .001	.80
Caseness of depression, N (%)	1 (1)	18 (19)	< .001	.70
*Fatigue, mean (SD)*				
Physical level	7.1 (2.7)	11.3 (4.1)	< .001	1.20
Mental level	4.3 (1.4)	5.4 (1.9)	< .001	.66
Total level	11.4 (3.7)	16.7 (5.3)	< .001	1.15
Caseness of chronic fatigue, N (%)	4 (5)	37 (39)	< .001	.95
Body Image Scale, mean (SD) ^b^	3.3 (4.2)	7.1 (6.6)	< .001	.68
*Menopause-specific QoL, mean (SD)*				
Vasomotor^b^	2.4 (1.6)	2.8 (1.9)	.17	
Psychosocial	2.2 (1.2)	3.7 (1.5)	< .001	1.10
Physical	2.3 (0.8)	3.6 (1.2)	< .001	1.27
*Current hormone replacement N (%)*	25 (28)	28 (30)	.84	
Paired relation, N (%)	69 (77)	68 (73)	.49	
Currently sexually active, N (%)	58 (61)	33 (35)	< .001	.53
	***N= 70***	***N = 73***		
*Intimate Bond Measure, mean (SD)*				
Partners' care	29.0 (7.0)	25.7 (9.6)	.002	.39
Partners' control ^b^	5.1 (6.8)	6.7 (7.2)	.094	
	***N = 54***	***N = 33***		
*Sexually active, mean (SD)*				
Pleasure^b^	8.9 (6.7)	5.8 (5.6)	.004	.50
Discomfort^b^	3.8 (1.8)	3.7 (1.9)	.88	

^a^Effect size. ^b^Non-parametric test

#### Stepwise hierarchical linear and logistic regression analyses

Relevant independent variables were grouped and entered in a stepwise hierarchical linear and logistic regression analysis to examine their association with the dimensional and categorical SRH score, respectively. Their association with the dimensional SRH and with the categorical SRH score is shown in Table 4. By using dimensional SRH the somatic complaints contributed mostly (39%) to the explained variance of the SRH score (p < .001). The demographic variables explained 19% of the variance in SRH (p < .001), physical or mental impairment contributed 3% (p < .001) and anxiety and fatigue contributed 3% (p = 0.01). Cancer-related variables (FIGO stage, relapse < 6 months, chemotherapy), use of medication, life style and menopausal variables did not make any significant contributions to the explained variance of the SRH score. In total the eight step model explained 70% of the variance in SRH (Table 4).

**Table 4:** Stepwise hierarchical linear and logistic regression analyses of variables associated to self rated health (SRH) score.

Independent variables	Dimensional SRH		Categorical SRH		
	*Beta*	*P*	*OR*	*95%CI*	*P*
*Step 1. Demography*					
Age at diagnosis	0.14	0.03	0.95	0.88–1.03	0.24
Civil status (Paired = reference)	0.05	0.34	1.00	0.13–7.43	0.99
Level of education (> 12 years = ref)	0.06	0.55	2.55	0.45–14.51	0.29
Work status (active reference)	-0.12	0.06	0.65	0.11–3.89	0.64
**Explained variance (R^2^)**	**0.187**	**< 0.001**	**0.084**		**0.055**

*Step 2. Cancer related variables*					
FIGO stage (I+II reference)	0.09	0.17	0.23	0.05–1.10	0.07
Relapse < 6 months (no = ref)	-0.05	0.39	0.53	0.09–3.15	0.49
Chemotherapy ever (no = ref)	-0.04	0.52	2.65	0.45–15.81	0.28
**Explained variance (R^2^)**	**0.218**	**0.15**	**0.109**		**0.424**
**Increase of variance (R^2^-change)**	**0.032**		**0.025**		

*Step 3. Somatic complaints*					
Nausea	-0.16	0.02	1.08	0.99–1.17	0.10
Pain	-0.15	0.05	1.05	1.01–1.09	0.01
Dyspnoe	-0.13	0.06	1.01	0.98–1.04	0.56
Insomnia	-0.08	0.32	1.01	0.96–1.02	0.52
Lack of appetite	-0.04	0.58	0.99	0.98–1.07	0.40
Constipation	-0.09	0.16	1.02	0.99–1.05	0.20
Diarrhea	-0.02	0.69	1.00	0.97–1.04	0.87
**Explained variance (R^2^)**	**0.606**	**< 0.001**	**0.571**		**< 0.001**
**Increase of variance (R^2^-change)**	**0.387**		**0.462**		

*Step 4. Impairment*					
Physical impairment	-0.10	0.16	11.73	1.28–108.02	0.03
Mental impairment	-0.08	0.22	0.53	0.05–5.39	0.57
**Explained variance (R^2^)**	**0.639**	**0.004**	**0.645**		**0.001**
**Increase of variance (R^2^-change)**	**0.033**		**0.074**		

*Step 5. Medication last year*					
Daily use of medication	-0.09	0.12	2.66	0.38–18.60	0.33
Use of analgetics	0.01	0.90	2.84	0.33–24.57	0.34
Use of psychotropics	-0.05	0.50	13.78	2.07–91.85	0.01
**Explained variance (R^2^)**	**0.653**	**0.17**	**0.717**		**0.001**
**Increase of variance (R^2^-change)**	**0.014**		**0.072**		

*Step 6. Anxiety and fatigue*					
HADS-Anxiety	-0.07	0.35	1.07	0.85–1.35	0.58
FQ Total fatigue	-0.16	0.02	1.24	1.05–1.47	0.01
**Explained variance (R^2^)**	**0.682**	**0.006**	**0.759**		**0.007**
**Increase of variance (R^2^-change)**	**0.029**		**0.042**		

*Step 7. Life style*					
Body Image Scale score	-0.12	0.07	1.09	0.92–1.30	0.32
BMI	0.01	0.83	0.97	0.80–1.18	0.79
Physical activity (moderate or more = reference)	-0.11	0.04	0.84	0.16–4.42	0.84
Smoking (no = reference)	0.04	0.54	0.67	0.08–5.49	0.71
**Explained variance (R^2^)**	**0.703**	**0.09**	**0.766**		**0.806**
**Increase of variance (R^2^-change)**	**0.021**		**0.007**		

*Step 8. Menopausal QoL*					
Vasomotor score	-0.08	0.14	1.26	0.78–2.03	0.35
**Explained variance (R^2^)**	**0.709**	**0.14**	**0.769**		**0.34**
**Increase of variance (R^2^-change)**	**0.006**		**0.003**		

By using stepwise logistic regression analyses of variables associated with the categorical SRH score, somatic complains contributed 46% of the explained variance of the SRH score (p < .001). The demographic variables explained 8% of the variance in SRH (p < .05), physical or mental impairment contributed 7% (p = .001) and anxiety and fatigue contributed 3% (p = .001). Cancer-related variables (FIGO stage, relapse < 6 months, chemotherapy), use of medication, life style and menopausal variables did not make any significant contributions to the explained variance of the categorical SRH score. In total the eight step model explained 77% of the variance in SRH.

#### Comparison of EOCSs and NORMs

**Figure 1:**
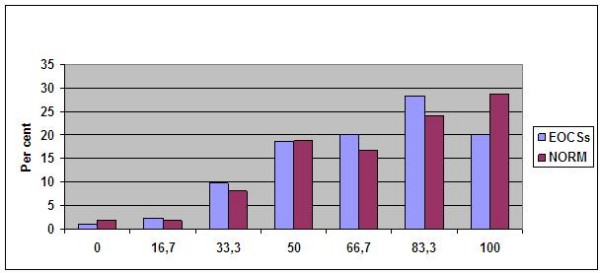
**Distribution of Q-29 scores in EOCSs and NORM**. Y- axis: Percent of EOCSs and NORM in relation to levels of SRH score.

No significant differences were found in level of education, paired relation, use of analgesics, psychotropics, HRT or antihypertensive drugs between EOCSs and NORM (data not shown). No significant differences in the overall distribution of the SRH scores between the EOCSs and NORM were observed, however a higher proportion of controls recorded a SRH score of 100 (p < .02) (Figure 1).

## Discussion

### Main findings

Somatic complaints (nausea, pain, dyspnoea, insomnia, lack of appetite, constipation), mental distress, fatigue, body image and menopause-related QoL, were significantly more common among EOCSs with "poor" rather than "good" SRH when using the median score to separate the two groups. Item #29 of the EORTC QLQ C-30 was thus strongly associated with common somatic complaints, which explained 39% of the variance using dimensional SRH and 46% using categorical SRH, but also with age, physical impairment and mental distress which explained a small but significant association. The 7% difference in explained variance using dimensional or categorical SRH and the different significance of pain, physical symptoms and prior use of psychotropic medicaments is important and interesting, but difficult to explain. The main interpretation of both models show a significant and important contribution for symptoms which appears to have clinical implications. The relevance of this single item in EOCSs may be supported by the fact that these symptoms are known to be frequently present among EOCSs, and are all conditions which may be accessible for therapeutic interventions. Interestingly the cancer-related variables (FIGO stage, recurrence within 6 months, chemotherapy ever), use of medication, life style and menopausal variables did not make any significant contributions to the explained variance of the SRH score in EOCSs used both as dimensional and categorical dependent variable. We did not observe any significant differences in the overall distribution of the SRH scores between the EOCSs and NORM, however, it was a difference in the highest score level, as a significantly higher proportion of controls recorded a SRH score of 100 (Figure 1). This indicates an important difference, indeed.

#### Comparison with other studies of SRH

SRH has not been studied in EOCSs previously. The use of a single – item measure of SRH has proven to have clinical value in primary care [8,12,17], as we also confirmed in our study. In accordance to the studies of Butt et al. [8] and Shadbolt et al. [12] in mixed samples of cancer patients, we found that a single question concerning SRH could be helpful for health care professionals to screen for especially physical problems in EOCSs, but also fatigue and mental distress. Compared to studies of general population samples by Al-Windi [9] and Eriksson et al. [10], we found that SRH also was associated with demographic and multiple somatic and mental symptoms.

#### Clinical implications

SRH allows the respondents in a summarized form to express different aspects of their health which they consider relevant, and some aspects of which may be accessible for therapeutic intervention [7,8]. Somatic complaints showed the strongest associations with the SRH score. To ask for SRH last week may be a quick and useful question to screen for symptoms in EOCSs which thus can be recognized by health care professionals. Somatic complaints, but also impairment and fatigue, are frequently present among EOCSs whether they are heavily treated or not. They may experience constipation or insomnia associated to surgical menopause or worry; some may have nausea and bowel dysfunction due to bowel surgery as part of primary or relapse surgery. Those with stabile disease or progression often experience various somatic symptoms due to peritoneal and bowel cancer spread, side effects of medication, or related to repeated courses of chemotherapy over months. Opportunities for treatment may then be easily evaluated by the gynaecologist or the regular GPs and provide basis for development of new strategies for better medical follow-up treatment of EOCSs on somatic and mental morbidity, fatigue, sexual problems, and quality of life.

#### Strength and limitation

The 66% respondent rate in EOCSs is considered as quite good since many women were treated many years ago and some of the questionnaires contained sensitive familial and sexual issues. We have used internationally accepted instruments that are well validated and showing good psychometric properties in our study, except for the M-QOL sexual domain. We also have analyzed attrition data on the non-respondents. There were only few significant differences between the responding and non-responding EOCSs, which indicate that the findings could be valid for our total sample of EOCSs and more generally.

One limitation of our study is that the EOCSs were treated over a long time period (29 years), however treatment and follow-up protocols were considered to be quite similar during that time period. Furthermore, the phenomenon of "response shift", explained as how individuals integrate changes in their health state so that their internal standards, values, or concepts of quality of life are nearly unchanged from before, may be considered as a limitation [32], as studies have shown that patients with objectively "poor health" often valued their health status as almost normal based on response shift [32]. This fact may also influence the experience of pain and other somatic complaints [32], and may explain the lack of the overall differences we found between cases and controls.

The cross- sectional design has its limitation by the present knowledge about previous health status. The use of item #29 of the EORTC QLQ-C30 and the median score as the cut off of a clinical classification for "poor" and "good" SRH may be open for discussion, compared to the use of the variable as a continuous measure. However, we found that a cut-off for "poor" and "good" SRH was useful to identify those at highest risk of morbidities and complaints and decreased SRH in EOCSs.

## Conclusion

SRH was strongly associated to common somatic complaints, but also to impairment and fatigue, which are well known symptoms in EOCSs, but not to cancer-related variables as FIGO stage, recurrence < 6 months or chemotherapy ever. A single question concerning SRH last week might be a quick method to screen for symptoms in EOCSs, some of which may be accessible for therapeutic interventions and in addition to cancer – related questions.

## Abbreviations

EOCSs: Epithelial ovarian cancer survivors; SRH: Self- Rated Health; FIGO: The International Federation of Gynecology and Obstetrics; BO: Bilateral- oophorectomy; GPs: General practitioners; EORTC: European Organization and Treatment of Cancer; QLQ C-30: Quality of Life Questionnaire; NORM: Women in the general population; HADS: The Hospital Anxiety and Depression Scale; FQ: The Fatigue Questionnaire; BIS: The Body Image Scale; IBM: The Intimate Bond Measure; M-QOL: The Menopause-Specific Quality of Life Questionnaire; SAQ: The Sexual Activity Questionnaire; HRT: Hormone replacement therapy; ESs: effect sizes; R^2^-change: explained variance.

## Competing interests

The authors declare that they have no competing interests.

## Authors' contributions

AHL has contributed to conception, design, provision of study material and patients, collection and assembly of data, data analysis and interpretation and manuscript writing.

AD has contributed to conception, design, provision of study materials and patients, collection and assembly of data, data analysis and interpretation, financial and administrative support and manuscript writing. SDF has contributed to collection and assembly of data, data analysis and interpretation and manuscript writing. CT has contributed to provision of study materials and patients, administrative support and manuscript writing. AAD has contributed to conception, design, data analysis and interpretation and manuscript writing. All authors have contributed, read and approved the final manuscript.

## Pre-publication history

The pre-publication history for this paper can be accessed here:

http://www.biomedcentral.com/1471-2407/9/2/prepub
